# Projecting wheat demand in China and India for 2030 and 2050: Implications for food security

**DOI:** 10.3389/fnut.2022.1077443

**Published:** 2023-01-26

**Authors:** Khondoker Abdul Mottaleb, Gideon Kruseman, Aymen Frija, Kai Sonder, Santiago Lopez-Ridaura

**Affiliations:** ^1^Department of Agricultural Economics and Agribusiness, University of Arkansas, Fayetteville, AR, United States; ^2^Sustainable Agrifood Systems, International Maize and Wheat Improvement Center (CIMMYT), Texcoco, Mexico; ^3^International Centre for Agricultural Research in the Dry Areas (ICARDA), Tunis, Tunisia

**Keywords:** aggregate demand, wheat, projection, time series, China, India

## Abstract

**Introduction:**

The combined populations of China and India were 2.78 billion in 2020, representing 36% of the world population (7.75 billion). Wheat is the second most important staple grain in both China and India. In 2019, the aggregate wheat consumption in China was 96.4 million ton and in India it was 82.5 million ton, together it was more than 35% of the world's wheat that year. In China, in 2050, the projected population will be 1294–1515 million, and in India, it is projected to be 14.89–1793 million, under the low and high-fertility rate assumptions. A question arises as to, what will be aggregate demand for wheat in China and India in 2030 and 2050?

**Methods:**

Applying the Vector Error Correction model estimation process in the time series econometric estimation setting, this study projected the per capita and annual aggregate wheat consumptions of China and India during 2019-2050. In the process, this study relies on agricultural data sourced from the Food and Agriculture Organization of the United States (FAO) database (FAOSTAT), as well as the World Bank's World Development Indicators (WDI) data catalog. The presence of unit root in the data series are tested by applying the augmented Dickey-Fuller test; Philips-Perron unit root test; Kwiatkowski-Phillips-Schmidt-Shin test, and Zivot-Andrews Unit Root test allowing for a single break in intercept and/or trend. The test statistics suggest that a natural log transformation and with the first difference of the variables provides stationarity of the data series for both China and India. The Zivot-Andrews Unit Root test, however, suggested that there is a structural break in urban population share and GDP per capita. To tackle the issue, we have included a year dummy and two multiplicative dummies in our model. Furthermore, the Johansen cointegration test suggests that at least one variable in both data series were cointegrated. These tests enable us to apply Vector Error Correction (VEC) model estimation procedure. In estimation the model, the appropriate number of lags of the variables is confirmed by applying the “varsoc” command in Stata 17 software interface. The estimated yearly per capita wheat consumption in 2030 and 2050 from the VEC model, are multiplied by the projected population in 2030 and 2050 to calculate the projected aggregate wheat demand in China and India in 2030 and 2050. After projecting the yearly per capita wheat consumption (KG), we multiply with the projected population to get the expected consumption demand.

**Results:**

This study found that the yearly per capita wheat consumption of China will increase from 65.8 kg in 2019 to 76 kg in 2030, and 95 kg in 2050. In India, the yearly per capita wheat consumption will increase to 74 kg in 2030 and 94 kg in 2050 from 60.4 kg in 2019. Considering the projected population growth rates under low-fertility assumptions, aggregate wheat consumption of China will increase by more than 13% in 2030 and by 28% in 2050. Under the high-fertility rate assumption, however the aggregate wheat consumption of China will increase by 18% in 2030 and nearly 50% in 2050. In the case of India, under both low and high-fertility rate assumptions, aggregate wheat demand in India will increase by 32-38% in 2030 and by 70-104% in 2050 compared to 2019 level of consumption.

**Discussions:**

Our results underline the importance of wheat in both countries, which are the world's top wheat producers and consumers, and suggest the importance of research and development investments to maintain sufficient national wheat grain production levels to meet China and India's domestic demand. This is critical both to ensure the food security of this large segment of the world populace, which also includes 23% of the total population of the world who live on less than US $1.90/day, as well as to avoid potential grain market destabilization and price hikes that arise in the event of large import demands.

## 1. Introduction

The world population is expected to reach from 8.9 to 10.6 billion by 2050, compared with 7.75 billion in 2020 (1), and an estimated 68% of those people will reside in urban areas, up from 61% in 2020 (1). The average per capita GDP at constant 2015 prices is projected to increase from US $11,33 in 2022 to US $13,747 in 2032 (2). The expected changes in demography, increases in per capita GDP, and greater urbanization will have major implications on future demand for foods and major cereals, such as wheat, maize, and rice. Households in countries in the early stages of development tend to consume a high proportion of cereals and coarse grains, which are relatively cheap sources of dietary energy (3, 4). As development progresses, with increases in income and urbanization, households tend to increase their consumption of energy-dense and more expensive foodstuffs, in place of cereals (4–7), a phenomenon described as the “nutrition transition” (5, 8–14). This paper focuses on how projected economic and demographic transitions, followed by nutrition transition, will affect future demand for wheat.

Several studies project global demand changes for major cereals and agricultural commodities, considering mainly demographic shifts. Such projections are highly relevant to target investments for addressing hunger and poverty. Predictions regarding global food demand, for example, foresee increases by 2050 that range from 70% (15) to 110% (16). Ray et al. (17) cautioned that, to meet rising demand, the average annual yield growth rates for major agricultural commodities, including wheat, should be at least 2.4% over 2005 levels. After reviewing 57 global food security projections, Van Dijk et al. (18) concluded that global food demand is expected to increase from 35 to 56% by 2050, over 2010 levels.

All the preceding are global studies, but GDP growth rate, urbanization, and demographic changes are heterogeneous across countries, and cropping and dietary intake patterns are country and region-specific. For example, in 2019, yearly per capita wheat consumption in Tunisia was nearly 198.4 kg, whereas, in Laos, it was only 1.4 kg (19). Country- and commodity-specific case studies can help to elucidate consumption directions for specific commodities, providing focused and important insights to target investments and policies.

This study examines evolving per capita consumption and aggregate demand for wheat in China and India projected to 2030 and 2050. Economic growth in those countries, measured in per capita GDP growth rate, has been among the fastest in the world, at an annua average l 8.3% for China and 4.2% for India, over 1990–2020 (1). The nominal per capita GDP of China increased from less than US $318 in 1990 to US $12,556 and, in India, from US $303 in 1990 to US $2,227 in 2020 (1). There are also important demographic changes underway in both countries. In 2021, the respective populations of China and India were 1.41 billion and 1.39 billion (1). Under the low fertility rate assumption, the total population of China is projected to be 1.44 billion in 2030 and 1.29 billion in 2050 whereas, assuming a high fertility rate China's population will be 1.49 billion in 2030 and 1.51 billion in 2050 (20). For India, low fertility rate projections foresee a population of 1.48 billion in 2030 and 1.49 billion in 2050 and, under a high fertility rate assumption, of 1.54 billion in 2030 and 1.79 billion in 2050 (20).

Urbanization has also accelerated in both countries. From a little more than one-quarter of China and India's populace living in cities in 1990 (1), by 2030, it is expected that nearly 71% of China's inhabitants and 40% of those in India will reside in urban areas (21) and, by 2050, four of every five persons in China and more than half of those in India will by city dwellers (21).

The dramatic changes in per capita GDP, population, and urbanization provide a unique opportunity to examine the effects of these factors on the future demand for wheat, a major food crop for both nations. China and India are the top wheat-producing and consuming countries in the world. In 2019, China produced 134.3 million t of wheat from 23.4 million ha, making it the world's number-one wheat producer and the third-highest in wheat area (22). That same year, India allocated 31.4 million ha of land to wheat—the world's largest area—and produced 105.6 million tons (t) of grain, the second-highest output (22). An estimated 36.5 million farms in China and 37.3 million in India grew wheat in 2020 (23), making the crop a major source of livelihood for farm households. An examination of the future of wheat demand can inform effective policies to improve the livelihoods of resource-poor wheat farmers in China and India.

This information can also help target policies to improve the food security and nutrition of the countries' inhabitants, who constitute 36% of world population of which 23% (22.5% in India and 0.5% in China) live on less than US $1.90/day (24), many of whom rely on wheat for dietary energy intake. In 1961, the yearly per capita total wheat consumption was less than 21 kg in China and 28 kg in India, which contributed to 12% of the per capita daily total calorie intake in China, and in India it was 11.8% (25). As of 2019, yearly per capita wheat consumption in China had increased to 65.8 kg, supplying 576 kcal per capita per day, and representing 17.2% of daily dietary energy, and in India to 60.4 kg, supplying 515 kcal per capita per day or 20% of daily dietary energy (19). In China and India, wheat is also a major source of protein. In 2019, the daily total protein intake per person in China was 105.1 grams, in which the contribution of wheat was 18.5 grams (17.6%) (19). In India, in the same year, the daily total protein intake per person is 64.9 grams, in which the contribution of wheat was 15.1 grams (23.2%) (19). Finally, despite being the top ranked wheat producing country, China is a net importer of wheat, and India exports sporadically. Thus, projections regarding their future wheat demand have clear significance, both with regards to internal effects and policies, as well as possible impacts on global wheat supplies and prices. In 2019, the countries jointly consumed 179 million t of wheat which was 35.4% of the total wheat (505 million t) consumed globally (19), net imports of wheat grain by China were 9.6 million t worth US $2.7 billion and India's net wheat export was 928.5 thousand t, worth US $243 million (26).

There has recently been global concern around ensuring diversified diets to nurture human health and the environment (27). Also, considering the importance of balanced diets to combat malnutrition and non-communicable disease, there is a call to increase investments in non-cereal crops, such as lentils and vegetables (5, 14). Based on those suggestions, if China and India loosen their efforts to produce more wheat and eventually started importing wheat from the global market, it can generate severe havoc on international wheat prices and food security of the wheat import-dependent developing countries.

The next section presents a brief literature review and the conceptual framework. Section 3 includes materials and methods, and Section 4 presents the major findings. Finally, Section 5 presents the conclusion and policy implications.

## 2. Literature review and the conceptual framework

Since its domestication around 10,000 years ago (28, 29), wheat has been playing a crucial role in ensuring food and nutrition securities in the world (30–32). In 2019, the yearly per capita wheat consumption in the world was around 66 kg, which supplied daily 538 kcal per person or 18% of total calorie intake (2,963 kcal), as well as 19.5% (16.3 grams) of the daily per capita protein (25). The adoption of improved agronomic practices and high-yielding wheat varieties have contributed significantly to increasing productivity in China, India, and worldwide (33–39). Currently, wheat is the most widely cultivated crop in the world, being grown on 219 million ha in 2020 (22). In 2020, global wheat production was worth nearly US $190 billion (40).

This study projects wheat demand in China and India during 2019–2050 and specifically focuses on 2030 and 2050. In terms of production and consumption, China and India are ranked as the first and second-largest wheat-producing countries respectively. In 2019, the global wheat production was more than 765 million t, of which 66% (505 million t) was used as food, and China and India jointly consumed 35.4% (179 million t) of the total wheat consumed globally in that year (25). Despite being the top wheat-producing countries, China is a net wheat importing country, and India exports sporadically. As the population of these countries is projected to increase by 2050, and as wheat is the second most important staple in China and India, it is imperative to examine the future demand for wheat to formulate investment strategies to ensure the food security of China and India.

There has recently been global concern around warranting varied diets to foster human health and the environment (27). Also, considering the importance of balanced diets to combat malnutrition and non-communicable disease, there is a call to increase investments in noncereal crops, such as lentils and vegetables (5, 14). Now, if China and India started reducing investment in major cereals, and if the demand for major cereals increases due to the increase in population, these countries will rely more on imports to meet their demand. However, if China and India started importing wheat in bulk from the international market, it can generate severe havoc on international wheat prices and food security of the wheat import-dependent developing countries.

Few studies have projected wheat demand in China and India. Rozelle and Huang (41) considered low- and high-income growth trends to conclude that, by 2020, yearly per capita wheat consumption in China lie between 80 and 83 kg. Applying the household model estimation procedure and employing primary data, Carter and Zhong (42) calculated a negative income elasticity for wheat and concluded that, in China, per capita consumption might decline, as later occurred it was 77 kg in 1990 and fell to 64 kg by 2018 (19).

For India, Chand (43), estimated a yearly per capita wheat consumption of 49.8 kg and an aggregate consumption of 67.5 million t in 2020–21. Mittal (44) projected that, in 2026, a yearly per capita wheat consumption of 48.9 kg and an aggregate consumption of 65.9 million t. Kumar et al. (45) forecast a 2021–22 yearly per capita wheat consumption of 47.6 kg and an aggregate consumption of 73.5 million t. Applying the QUAIDS (Quadratic Almost Ideal Demand System) estimation procedure, Ganesh-Kumar et al. (46) estimated a negative (-0.13) expenditure elasticity for wheat and projected the yearly per capita wheat consumption at 49.3 kg by 2026 and an aggregate wheat consumption ranging from 63.3 to 69.4 million t, dependent on income growth rate assumptions. By 2018, India's yearly wheat consumption surpassed projections considerably, with a per capita consumption of 62 kg and an aggregate wheat consumption of 83.5 million t (19). So, demand forecasts have fallen short to date, suggesting the need to revisit this issue using innovative methods and models.

Several studies have documented wheat consumption growth for countries in sub-Saharan Africa and South Asia, because of rising per capita GDP and urbanization (4, 47, 48). In the present study, the long-term influence of the share of the urban population, domestic wheat production, GDP per capita, and wheat imports are considered to estimate future per capita wheat consumption in China and India. We applied the Vector Error Correction (VEC) estimating procedure under the time series estimation setting. Finally, employing the Box-Jenkins methods for forecasting (49), we forecasted yearly per capita wheat consumption in China and India, providing up-to-date estimates through a simple but strong econometric estimation procedure using the most recent datasets.

## 3. Materials and methods

This study relies on agricultural data sourced from the Food and Agriculture Organization of the United States (FAO) database (FAOSTAT), as well as the World Bank's World Development Indicators (WDI) data catalog. Data on yearly per capita wheat consumption in kg (*PC*_*t*_), imports (*IMP*_*t*_), and domestic production (*DPR*_*t*_), yields, and the wheat area were retrieved from FAO online datasets (FAOSTAT). Data for the percentage of urban population (*UR*_*t*_) and per capita GPD in current US$ (*GDP*_*t*_) were collected from the World Bank's World Development Indicators (WDI) catalog.

The study's equations of interest are specified below:


(1) $$\begin{array}{l} {△lnPC}_{t}=\sigma +\sum_{i=1}^{k}\beta_{i}{△lnPC}_{t-i}+\sum_{j=1}^{k}\alpha_{j}{△ln\%UR}_{t-j}\\ +\sum_{l=1}^{k}\phi_{j}{△lnGDP}_{t-l}+\sum_{m=1}^{k}\gamma_{m}{△lnDPR}_{t-m}\\ +\;\sum_{n=1}^{k}\theta_{n}{△lnIMP}_{t-n}+\psi yd82\\ +\sum_{n=1}^{k}\tau_{n}\left(yd82\;\times {△ln\%UR}_{t-j}\right)\\ +\sum_{n=1}^{k}\omega_{n}\left(yd82\;\times {△lnGDP}_{t-l}\right)+\lambda_{1}{ECT}_{t-1}+\mu_{1\text{t}}\\ {△ln\%UR}_{t}=\varphi +\sum_{i=1}^{k}\beta_{i}{△lnPC}_{t-i}+\sum_{j=1}^{k}\alpha_{j}{△ln\%UR}_{t-j}\\ +\sum_{l=1}^{k}\phi_{j}{△lnGDP}_{t-l}+\sum_{m=1}^{k}\gamma_{m}{△lnDPR}_{t-m}\\ +\;\sum_{n=1}^{k}\theta_{n}{△lnIMP}_{t-n}+\psi yd82\\ +\sum_{n=1}^{k}\tau_{n}\left(yd82\;\times {△ln\%UR}_{t-j}\right)\\ +\sum_{n=1}^{k}\omega_{n}\left(yd82\;\times {△lnGDP}_{t-l}\right)+\lambda_{2}{ECT}_{t-1}+\mu_{2\text{t}}\\ {△lnGDP}_{t}=\tau +\sum_{i=1}^{k}\beta_{i}{△lnPC}_{t-i}+\sum_{j=1}^{k}\alpha_{j}{△ln\%UR}_{t-j}\\ +\sum_{l=1}^{k}\phi_{j}{△lnGDP}_{t-l}+\sum_{m=1}^{k}\gamma_{m}{△lnDPR}_{t-m}\\ +\;\sum_{n=1}^{k}\theta_{n}{△lnIMP}_{t-n}+\psi yd82\\ +\sum_{n=1}^{k}\tau_{n}\left(yd82\;\times {△ln\%UR}_{t-j}\right)\\ +\sum_{n=1}^{k}\omega_{n}\left(yd82\;\times {△lnGDP}_{t-l}\right)+\lambda_{3}{ECT}_{t-1}+\mu_{3\text{t}}\\ {△lnDPR}_{t}=\tau +\sum_{i=1}^{k}\beta_{i}{△lnPC}_{t-i}+\sum_{j=1}^{k}\alpha_{j}{△ln\%UR}_{t-j}\\ +\sum_{l=1}^{k}\phi_{j}{△lnGDP}_{t-l}++\;\sum_{n=1}^{k}\theta_{n}{△lnIMP}_{t-n}\\ +\;\psi yd82+\sum_{n=1}^{k}\tau_{n}\left(yd82\;\times {△ln\%UR}_{t-j}\right)\\ +\sum_{n=1}^{k}\omega_{n}\left(yd82\;\times {△lnGDP}_{t-l}\right)\\ +\lambda_{4}{ECT}_{t-1}+\mu_{4\text{t}}\\ {△lnIMP}_{t}=\upsilon +\sum_{i=1}^{k}\beta_{i}{△lnPC}_{t-i}+\sum_{j=1}^{k}\alpha_{j}{△ln\%UR}_{t-j}\\ +\sum_{l=1}^{k}\phi_{j}{△lnGDP}_{t-l}+\sum_{m=1}^{k}\gamma_{m}{△lnDPR}_{t-m}\\ +\;\sum_{n=1}^{k}\theta_{n}{△lnIMP}_{t-n}+\psi yd82\\ +\sum_{n=1}^{k}\tau_{n}\left(yd82\;\times {△ln\%UR}_{t-j}\right)\\ +\sum_{n=1}^{k}\omega_{n}\left(yd82\;\times {△lnGDP}_{t-l}\right)+\lambda_{5}{ECT}_{t-1}+\mu_{5\text{t}}\\ yd82=\upsilon +\sum_{i=1}^{k}\beta_{i}{△lnPC}_{t-i}+\sum_{j=1}^{k}\alpha_{j}{△ln\%UR}_{t-j}\\ +\sum_{l=1}^{k}\phi_{j}{△lnGDP}_{t-l}+\sum_{m=1}^{k}\gamma_{m}{△lnDPR}_{t-m}\\ +\;\sum_{n=1}^{k}\theta_{n}{△lnIMP}_{t-n}\\ +\sum_{n=1}^{k}\tau_{n}\left(yd82\;\times {△ln\%UR}_{t-j}\right)\\ +\sum_{n=1}^{k}\omega_{n}\left(yd82\;\times {△lnGDP}_{t-l}\right)\\ +\lambda_{6}{ECT}_{t-1}+\mu_{6\text{t}}\\ \left(yd82\;\times {△ln\%UR}_{t-j}\right)=\upsilon +\sum_{i=1}^{k}\beta_{i}{△lnPC}_{t-i}+\sum_{j=1}^{k}\alpha_{j}{△ln\%UR}_{t-j}\\ +\sum_{l=1}^{k}\phi_{j}{△lnGDP}_{t-l}+\sum_{m=1}^{k}\gamma_{m}{△lnDPR}_{t-m}\\ +\;\sum_{n=1}^{k}\theta_{n}{△lnIMP}_{t-n}+\psi yd82\\ +\sum_{n=1}^{k}\tau_{n}\left(yd82\;\times {△ln\%UR}_{t-j}\right)\\ +\sum_{n=1}^{k}\omega_{n}\left(yd82\;\times {△lnGDP}_{t-l}\right)+\lambda_{7}{ECT}_{t-\;1}+\mu_{7\text{t}}\\ \left(yd82\;\times {△lnGDP}_{t-l}\right)=\upsilon +\sum_{i=1}^{k}\beta_{i}{△lnPC}_{t-i}+\sum_{j=1}^{k}\alpha_{j}{△ln\%UR}_{t-j}\\ +\sum_{l=1}^{k}\phi_{j}{△lnGDP}_{t-l}+\sum_{m=1}^{k}\gamma_{m}{△lnDPR}_{t-m}\\ +\;\sum_{n=1}^{k}\theta_{n}{△lnIMP}_{t-n}+\psi yd82\\ +\sum_{n=1}^{k}\tau_{n}\left(yd82\;\times {△ln\%UR}_{t-j}\right)\\ +\sum_{n=1}^{k}\omega_{n}\left(yd82\;\times {△lnGDP}_{t-l}\right)+\lambda_{8}{ECT}_{t-1}+\mu_{8\text{t}} \end{array}$$

Where:

**Table:** 

*lnPC*	= Natural log of yearly per capita wheat consumed;
*ln%UR*_*t*_	= Natural log of the share of urban population (%);
*lnGDP*_*t*_	= Natural log of the per capita GDP (US$);
*lnDPR*_*t*_	= Natural log of the domestically produced wheat in tons; and
*lnIMP*_*t*_	= Natural log of wheat imported (tons)
*yd*82	= Year>1981 dummy (yes=1)
k-1	= the lag length is reduced by 1
β_*i*_, α_*j*_, ϕ_*j*_, γ_*m*_, θ_*n*_, ψ, τ, ω	= short-run dynamic coefficients of the model's adjustment toward long-term equilibrium
**λ**_**i**_	**=** speed of adjustment parameter with a negative sign;
***ECT_t−1_***	= the error correction term is the lagged value of the residuals obtained from the cointegration regression of the dependent variables on the regressors. Contains long-term information derived from the long-term cointegration relationship;
**μ**_**it**_	**=** the residuals (stochastic error term/ impulses or innovations or shocks.

Before estimating Equation (1), as parts of estimation process, the presence of unit root in the data series are tested by applying the augmented Dickey-Fuller test. However, as it is argued that the augmented Dickey-Fuller test is outdated, we also have employed, Philips-Perron unit root test; Kwiatkowski-Phillips-Schmidt-Shin test, and Zivot-Andrews Unit Root test allowing for a single break in intercept and/or trend. The results from these tests are included in the Annexure. The test statistics suggest that a natural log transformation and with the first difference of the variables provides stationarity of the data series for both China and India. The Zivot-Andrews Unit Root test, however, suggested that there is a structural break in urban population share and GDP per capita. To handle the issue, we have included a year dummy and two multiplicative dummies in our model. Furthermore, the Johansen cointegration test suggests that at least one variable in both data series were cointegrated. These tests enable us to apply Vector Error Correction (VEC) model estimation procedure. In estimation the model, the appropriate number of lags of the variables is confirmed by applying the “*varsoc”* command in Stata 17 software interface. Based on the test statistics, we have applied the VEC estimation procedure, which allow us to elucidate the long-term relationships among the yearly per capita wheat consumption and the variables of interest: the yearly per capita GDP, share of the urban population, and the domestic production of wheat and wheat import.

After estimating the VEC models, we applied the simple dynamic forecasting process, to forecast yearly per capita wheat consumption in China and India. Finally, to estimate the aggregate wheat demand in 2030 and 2050, we used the following process:


(2) $$\begin{array}{l} {\text{AWD}}_{\text{t}}=\hat{PC_{t}}\;\times \;PP_{t} \end{array}$$

Where:

AWD_t_ = Aggregate wheat demand in year t (= 2030 and 2050);
$\hat{PC_{t}}$ = Estimated yearly per capita wheat consumption (kg);
*PP*_*t*_ = Projected population in year t (= 2030 and 2050).

Using Equation (2), we estimated the aggregate wheat demand of China and India in 2030 and 2050 considering both changes in the per capita wheat consumption and in population.

## 4. Discussions and major findings

### 4.1. Descriptive findings

The worldwide trends of land allocation to wheat (million ha), wheat production (million t), yield (t/ha), consumption (kg/capita/year) and daily per capita calorie intake from wheat are during 1961–2020 are presented in Table 1. In 1961, at least 94 countries in the world cultivated wheat on at least on 204 million ha of land. The average land allocation per country was nearly 2.2 million ha, and with an average yield of 1.09 t/ha, total wheat production in the world was 222 million t (Table 1). In the same year, wheat was consumed by at least 154 countries, and the per capita yearly wheat consumption was nearly 55 kg, that supplied 415 kcal energy daily to a person which was nearly 19% of the total calorie intake of a person in 1961 (Table 1). In 2020, at least 124 countries in the world cultivated wheat on at least on 219 million ha of land. With an average yield of 3.47 t/ha, the total wheat production was 760 million t. In 2019, the yearly per capita wheat consumption was nearly 66 kg that supplied 538 kcal of daily dietary energy per person, which was more than 18% of the daily total calorie intake by a person (Table 1).

**Table 1:** Temporal changes in wheat production and consumption in the world (1961–2020).

**Year**	**1961**	**1971**	**1981**	**1991**	**2001**	**2011**	**2019/2020**
**Land allocation (million ha)**							
No. of countries	94	98	101	103	125	123	124
Area, average	2.17	2.18	2.37	2.18	1.72	1.79	1.77
Total area	204.2	213.9	239.2	224.2	214.6	220.3	219.0
Standard deviation	7.81	7.8	8.09	7.07	4.97	5.19	5.23
Minimum	0.00	0.00	0.00	0.00	0.00	0.00	0.00
Maximum	63	64	59.2	45.9	25.7	29.1	31.4
**Production (million t)**							
Production, average	2.37	3.55	4.45	5.32	4.71	5.67	6.14
Total production	222.4	347.5	449.6	547.8	588.2	696.9	760.1
Standard deviation	7.6	11.6	14.1	17	15.2	18.4	21.3
Minimum	0	0	0	0	0	0	0
Maximum	62.5	92.8	76.7	96	93.9	117.4	134.3
**Yield (t/ha)**							
Yield	1.09	1.62	1.88	2.44	2.74	3.16	3.47
Standard deviation	0.92	1.20	1.46	1.86	1.86	1.89	2.10
Minimum	0.22	0.33	0.40	0.20	0.37	0.39	0.40
Maximum	4.12	4.97	6.70	7.86	9.06	9.86	9.93
**Consumption (kg/capita/year)**							
No. of countries	154	154	154	155	175	175	180
Average consumption	54.9	56.5	65.0	70.8	68.8	65.3	65.9
Standard deviation	52.7	49.8	48.8	48.2	48.6	46.8	45.4
Minimum	0.0	1.6	1.4	0.1	1.6	1.9	1.4
Maximum	226.0	220.3	210.6	221.0	215.4	219.7	198.5
**Calorie intake (kcal/capita/day)**							
Calorie intake from wheat	415	436	515	569	555	526	538
Standard deviation	397	374	371	373	375	363	359
Minimum	0	11	11	0	13	15	18
Maximum	1,688	1,569	1,589	1,667	1,634	1,645	1,584
Daily total calorie intake (kcal/capita/day)	2,196	2,365	2,501	2,601	2,725	2,869	2,963
Share of wheat (%)	18.9	18.4	20.6	21.9	20.4	18.3	18.2

Source : FAOSTAT (26).

In Table 2, the temporal changes in land allocation to wheat, production, yield, and consumption, are presented for China and India. Wheat is the second most preferred staple after rice both in China and India. It shows that, while in China the land allocation to wheat had increased from 25.6 million ha in 1961 to 30.9 million ha in 1991, later it declined to almost the original size, at 23.4 million ha in 2020. In contrast, the land allocation to wheat has continuously increased in India since 1961. However, total wheat production in China and India has increased continuously since 1961, due mainly to rising yields (Table 2).

**Table 2:** Temporal changes in wheat area (million ha) production (million t), yield (t/ha) and consumption (kg and kcal) in China and India.

**Year**	**China**	**India**
**Land allocation to wheat (million ha)**		
1961	25.6	12.9
1971	25.6	18.2
1981	28.3	22.3
1991	30.9	24.2
2001	24.7	25.7
2011	24.3	29.1
2020	23.4	31.4
**Production (million t)**		
1961	14.3	11.0
1971	32.6	23.8
1981	59.6	36.3
1991	96.0	55.1
2001	93.9	69.7
2011	117.4	86.9
2020	134.3	107.6
**Yield (t/ha)**		
1961	0.56	0.85
1971	1.27	1.31
1981	2.11	1.63
1991	3.11	2.28
2001	3.80	2.71
2011	4.83	2.99
2020	5.74	3.43
**Consumption (capita/kg/year)**		
1961	20.9	27.9
1971	33.0	36.7
1981	62.6	45.6
1991	77.6	60.3
2001	71.9	62.2
2011	63.0	58.9
2019	65.8	60.4

Source :FAOSTAT (22).

Yearly per capita wheat consumption in China follows a pattern similar to that of land allocations to wheat: it increased initially until 1991 and later decreased slightly. In 1961, the yearly per capita wheat consumption in China was nearly 21 kg and increased to more than 77 kg in 1991 and, by 2019, declined to nearly 66 kg (Table 2). In contrast in India, yearly per capita wheat consumption has increased steadily from 28kg in 1961 to 60kg in 2019 (Table 2).

**Figure 1:**
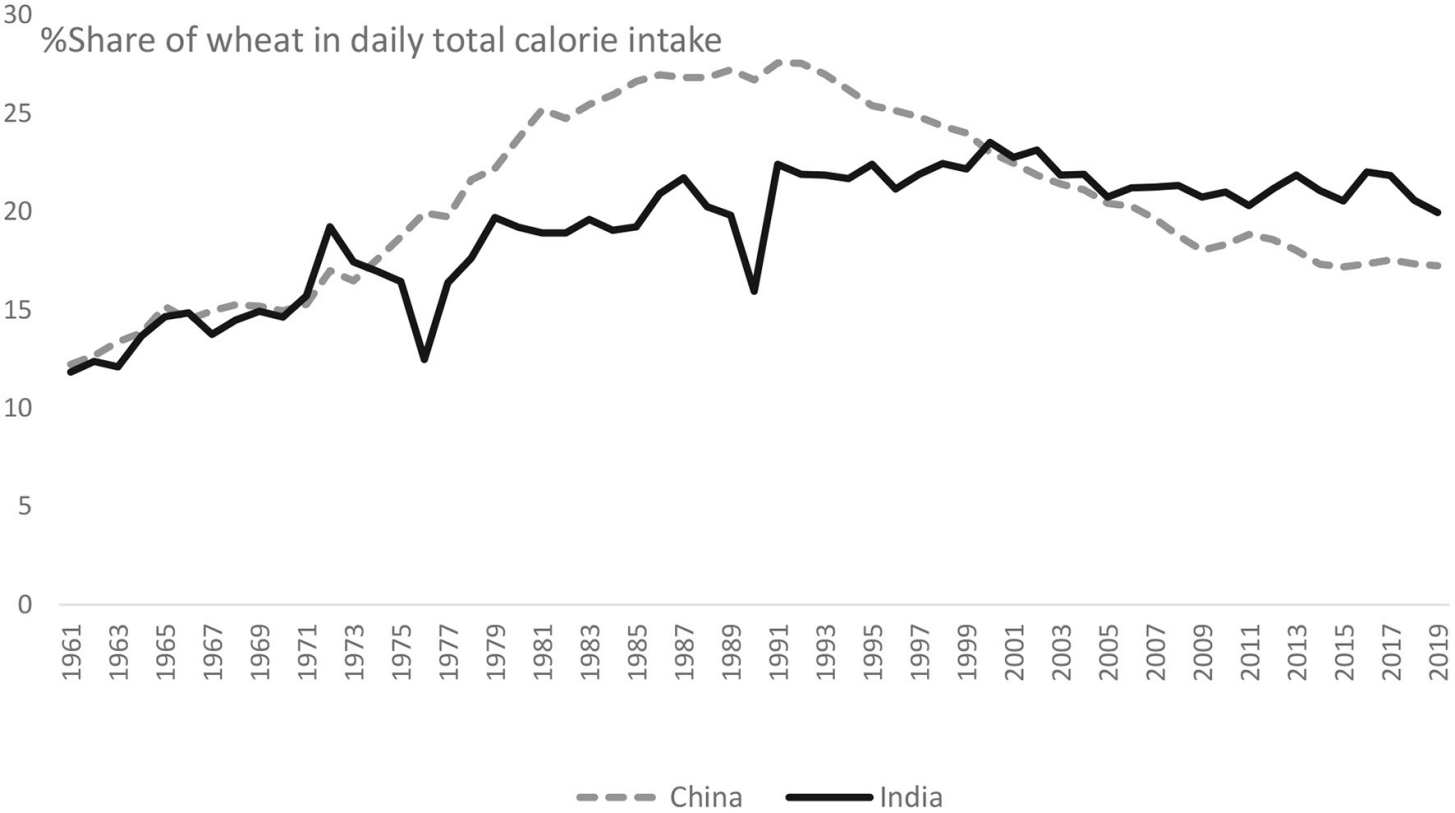
Share of wheat in daily total calorie intake in China and India during 1961–2019 [(per capita daily calorie intake from wheat ÷ Per capita daily total calorie intake) × 100]. Source: Authors, based on FAOSTAT (22).

Furthermore, the importance of wheat in safeguarding the daily dietary energy security in China and India has increased over the years. In 1961, the contribution of wheat to daily dietary energy in China was 176 kcal per capita, which was 12% of the daily total per capita calorie intake in China (Figure 1). In India, it was 238 kcal, which was <12% of per capita daily calorie intake (Figure 1). By 2019, the contribution of wheat to the daily calorie intake in China had risen to 17% and, in India, 20% (Figure 1).

**Figure 2:**
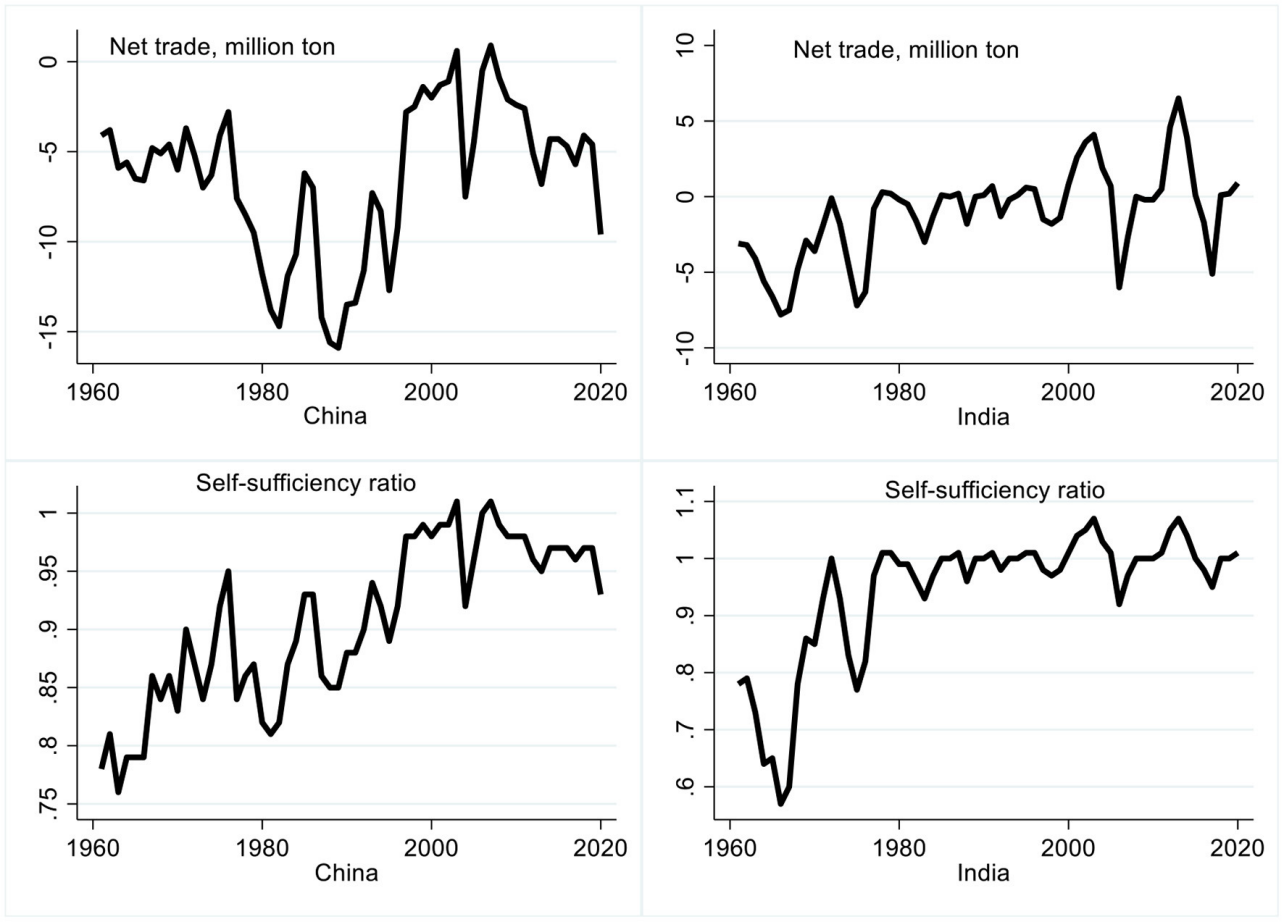
Net export (export-import) in million t and self-sufficiency status [domestic production ÷ (domestic production+import-export)] of China and India during 1961–2020. Source: Authors, based on FAOSTAT (22).

The temporal changes in net wheat trade (exports—imports) in million t, and self-sufficiency trends, which is measured as domestic production / [(domestic production + (import-export)] are presented for China and India during 1961–2020 in Figure 1. Only around year 2000 did both nations begin to export wheat, but only sporadically (Figure 2). An analysis of wheat self-sufficiency trends, show that despite dramatic increases in wheat yields, both countries can just meet their domestic demand and often rely on imports to satisfy demand spikes (Figure 2).

The economies of China and India are changing dynamically, due to changes in population, income, and urbanization. Under the United Nations (20) assumption of low fertility, the population in China may increase to 1.44 billion in 2030 and decline to 1.29 billion by 2050 and, under the assumption of high fertility, it could increase to 1.49 billion in 2030 and 1.52 billion in 2050 (Table 3). At the same time, 80% of China's inhabitants will reside in urban areas by 2050, compared to 61.4% in 2020, and the per capita GDP of China is projected to reach US $17,325 by 2030, compared to US $10,500 in 2020 (Table 3). Under both low and high fertility growth rate assumptions, the population of India is projected to increase by 2030 and 2050, possibly reaching 1.49 billion or 1.79 billion persons by mid-century, depending on the fertility rate assumption used (Table 3). Furthermore, by 2030 and 2050, more than 40% and 53% of inhabitants respectively will reside in urban areas, compared to 35% in 2020, and the projected GDP per capita will average US $3,079 by 2030, compared to US $1,900 in 2020 (Table 3).

**Table 3:** Temporal changes and projection of population (million), the share of the urban population, and GDP per capita (US $) 1961–2050.

								**Projected**			
	**1961**	**1971**	**1981**	**1991**	**2001**	**2011**	**2020**	**2030**	**2030**	**2050**	**2050**
**China**											
Population (million	660	841	994	1,151	1,272	1,344	1,402	1,437^a^	1,492^a^	1,294^a^	1,515^a^
Urban population (%)	17	17	20	27	37	51	61.4	70.6^b^		80.0^b^	
GDP per capita (US$)	141	238	360	786	1,901	4,961	10,500	17,325^c^			
**India**											
Population (million	460	568	715	891	1,075	1,250	1,380	1,468^a^	1,540^a^	1,489^a^	1,793^a^
Urban population (%)	18	20	23	26	28	31	35	40.1^b^		53.0^b^	
GDP per capita (US$)	336	394	438	576	852	1,410	1,900	3,079^c^			
**World**											
Population (million	3,072	3,761	4,511	5,368	6,194	7,003	7,753	8,363^a^	8,734^a^	8,907^a^	10,588^a^
Urban population (%)	34	37	40	43	47	52	56.2	60.4^b^		68.3^b^	
GDP per capita (US$)	3,865	5,340	6,322	7,177	8,223	9,739	10,926	13074^c^			

Sources: World Bank (1);

a United Nations (20);

b World Bank (21);

c Real GDP per capita in 2010 price USDA (2).

In the next section, we have econometrically estimated the yearly per capita wheat consumption in China and India in 2030 and 2050, considering long-term relationships among the per capita yearly wheat consumption, the GDP per capita, the share of the urban population, domestic production and import of wheat. Based on the projected wheat consumption, we have calculated the aggregate wheat demand for 2030 and 2050.

### 4.2. Econometric findings

The long-term relationship between China's yearly per capita wheat consumption and the variables of interest are detailed in Table 4.

**Table 4:** Estimated functions applying the Vector Error Correction (VEC) model estimation procedure, explaining the relationship between yearly per capita wheat consumption, wheat import, % share of the urban population, domestic wheat production, and GDP per capita in China.

**Dependent variables**	**d.ln(Cons)**	**d.ln(Imp)**	**d.ln(%Urb)**	**d.ln(Pro)**	**d.ln(GDP)**	**Year > 1981 dummy (y82)**	**y82 X ln(GDP)**	**y82 X ln(%Urb)**
ECT_t − 1_	−0.11 (0.11)	0.81 (1.69)	0.21^***^ (0.04)	0.37 (0.28)	−0.21 (0.14)	0.64^*^ (0.35)	3.62^*^ (2.11)	2.08^*^ (1.07)
D.ln(Cons)_t − 1_	−0.49^***^ (0.16)	−0.81 (2.51)	−0.0025 (0.06)	−0.19 (0.41)	0.47^**^ (0.20)	−0.70 (0.52)	−4.05 (3.12)	−2.19 (1.58)
D.ln(Imp)_t − 1_	−0.0024 (0.01)	−0.35^**^ (0.15)	−0.0019 (0.00)	0.026 (0.02)	0.0040 (0.01)	0.034 (0.03)	0.20 (0.18)	0.10 (0.09)
D.ln(%Urb)_t − 1_	0.023 (0.25)	1.06 (3.91)	−0.43^***^ (0.10)	−0.17 (0.64)	0.76^**^ (0.31)	1.99^**^ (0.81)	11.8^**^ (4.87)	6.10^**^ (2.47)
D.ln(Pro)_t − 1_	−0.071 (0.07)	0.19 (1.15)	0.061^**^ (0.03)	−0.26 (0.19)	−0.097 (0.09)	0.73^***^ (0.24)	4.26^***^ (1.43)	2.25^***^ (0.73)
D.ln(GDP) _t − 1_	0.18^*^ (0.11)	−2.03 (1.63)	−0.16^***^ (0.04)	0.83^***^ (0.27)	−0.039 (0.13)	−0.027 (0.34)	−0.16 (2.03)	−0.071 (1.03)
Year > 1981 dummy (y82)	−0.44 (0.43)	2.99 (6.64)	0.77^***^ (0.16)	1.38 (1.08)	−0.76 (0.53)	2.43^*^ (1.38)	7.91 (8.27)	4.85 (4.20)
D.y82 X ln(GDP) _t − 1_	0.080 (0.24)	−4.23 (3.73)	−0.13 (0.09)	−1.47^**^ (0.61)	0.76^**^ (0.30)	−0.26 (0.78)	−1.10 (4.65)	−0.65 (2.36)
D. y82 X ln(%Urb)_t − 1_	−0.15 (0.47)	8.23 (7.24)	0.26 (0.18)	2.89^**^ (1.18)	−1.48^**^ (0.58)	0.48 (1.51)	2.00 (9.02)	1.21 (4.58)
Constant	0.00028 (0.01)	−0.027 (0.10)	−0.0052^**^ (0.00)	−0.014 (0.02)	0.0048 (0.01)	0.00073 (0.02)	0.0087 (0.12)	−0.0012 (0.06)
No. of observations				55				
AIC				−26.06				
HQIC				−24.83				
SBIC				−22.89				
Log likelihood				803.7				
R^2^	0.41	0.26	0.74	0.45	0.22	0.27	0.63	0.63

Values in parentheses are standard errors.

* Significant at the 10% level.

** Significant at the 5% level.

*** Significant at the 1% level.

Cons = yearly per capita wheat consumption in kg.

Imp = wheat import in 1,000, ton.

%Urb = % Urban population.

Pro = domestic wheat production in 1,000 tons.

GDP =per capita GDP in nominal US $.

y82 =Year > 1981 dummy (yes=1).

The estimated error correction equation (ECT) for China is:


(3) $$\begin{array}{c} {ECT}_{t-1}=lnPC_{t-1}-+0.03{△lnIMP}_{t-1}-0.85△{ln\%UR}_{t-1}\\ -0.72△lnDPR_{t-1}-0.25△lnGDP_{t-1}\\ +0.005\;\left(Year>1981\;dummy\right)\\ +2.56\;\left(Year>1981\;dummy\;\times △lnGDP_{t-1}\right)\\ -6.53\;\left(Year>1981\;dummy\;\times △{ln\%UR}_{t-1}\right)+0.05 \end{array}$$

And, setting the yearly per capita wheat consumption (PC) as the target variable, the estimated per capita wheat consumption equation for China is as follows:


(4) $$\begin{array}{c} {△lnPC}_{t}=-0.11-0.49{△lnPC}_{t-1}-0.003\;{△lnIMP}_{t-1}\\ +\;0.02{△ln\%UR}_{t-1}-\;0.07{△lnDPR}_{t-1}\\ +0.18{△lnGDP}_{t-1}-0.44\;\left(year>1981\;dummy\right)\\ +0.08\;\left({year>1981\;dummy\times △lnGDP}_{t-1}\right)\\ -0.15\left({year>1981\;dummy\times △ln\%UR}_{t-1}\right)-0.0002 \end{array}$$

It is important to mention here is that in Equation (3), the sign of the estimated coefficients needs to explain in a reverse way [e.g., (50, 51)]. It shows that, in the long run, with other factors remaining the same, the share of urban population (*p* < 0.05), domestic wheat production (*p* < 0.00) and the share of urban population after 1981 will positively and significantly impact the long run yearly per capita wheat consumption in China. It means, with the increase in domestic wheat production, and increased people in the urban areas, the yearly per capita wheat consumption in China will increase in the long-run. Conversely, while an increase in GDP per capita (*p* < 0.00) will have a positive but insignificant impact on the yearly per capita wheat consumption in China the long-run, the increase in GDP per capita after 1981 will have negative and significant impact on the per capita wheat consumption in China (*p* < 0.00).

For India, the long-term relationship between India's yearly per capita wheat consumption, urban population (%), imports, domestic wheat production, and per capita GDP are presented in Table 5.

**Table 5:** Estimated functions applying the Vector Error Correction (VEC) model estimation procedure, explaining the relationship between yearly per capita wheat consumption, wheat import, % share of the urban population, domestic wheat production, and GDP per capita in India.

**Dependent variables**	**d.ln(Cons)**	**d.ln(Imp)**	**d.ln(%Urb)**	**d.ln(Pro)**	**d.ln(GDP)**	**Year > 1981 dummy (y82)**	**y82 X ln(GDP)**	**y82 X ln(%Urb)**
ECT_t − 1_	0.0055 (0.01)	0.39 (0.50)	0.0098^***^ (0.00)	0.024^**^ (0.01)	−0.0071^**^ (0.00)	0.032^**^ (0.01)	0.20^**^ (0.09)	0.10^**^ (0.04)
D.ln(Cons)_t − 1_	−0.60^***^ (0.12)	5.02 (4.07)	−0.021 (0.02)	−0.029 (0.09)	0.031 (0.03)	0.013 (0.11)	0.10 (0.69)	0.025 (0.36)
D.ln(Imp)_t − 1_	0.0021 (0.00)	−0.40^***^ (0.14)	−0.0013^**^ (0.00)	−0.0033 (0.00)	0.00074 (0.00)	0.00099 (0.00)	0.0059 (0.02)	0.0013 (0.01)
D.ln(%Urb)_t − 1_	0.58 (1.46)	16.0 (49.07)	0.28 (0.22)	1.53 (1.03)	−0.66^**^ (0.34)	2.55^*^ (1.37)	15.5^*^ (8.32)	7.99^*^ (4.34)
D.ln(Pro)_t − 1_	−0.14 (0.15)	−5.93 (4.89)	0.047^**^ (0.02)	−0.37^***^ (0.10)	−0.093^***^ (0.03)	0.18 (0.14)	1.09 (0.83)	0.59 (0.43)
D.ln(GDP) _t − 1_	−0.69 (0.88)	−40.0 (29.46)	−0.30^**^ (0.13)	0.98 (0.62)	−0.31 (0.20)	−1.30 (0.82)	−8.04 (4.99)	−4.04 (2.61)
Year > 1981 dummy (y82)	−0.86 (2.49)	−65.2 (83.72)	−1.67^***^ (0.38)	−3.97^**^ (1.75)	1.19^**^ (0.57)	−5.39^**^ (2.33)	−39.1^***^ (14.19)	−20.1^***^ (7.41)
D.y82 X ln(GDP) _t − 1_	0.059 (0.85)	−9.48 (28.73)	0.38^***^ (0.13)	1.12^*^ (0.60)	−0.28 (0.20)	1.31 (0.80)	7.97 (4.87)	4.32^*^ (2.54)
D. y82 X ln(%Urb)_t − 1_	−0.11 (1.65)	17.8 (55.47)	−0.73^***^ (0.25)	−2.13^*^ (1.16)	0.55 (0.38)	−2.54^*^ (1.54)	−15.4 (9.40)	−8.36^*^ (4.91)
Constant	−0.00059 (0.02)	−0.065 (0.67)	0.00012 (0.00)	−0.0016 (0.01)	0.0013 (0.00)	0.016 (0.02)	0.099 (0.11)	0.051 (0.06)
No. of observations				55				
AIC				−18.6				
HQIC				−17.4				
SBIC				−15.45				
Log likelihood				599.3				
R^2^	0.41	0.33	0.58	0.60	0.42	0.17	0.58	0.58

Values in parentheses are standard errors.

* Significant at the 10% level.

** Significant at the 5% level.

*** Significant at the 1% level.

Cons = yearly per capita wheat consumption in kg.

Imp = wheat import in 1,000 ton.

%Urb = % Urban population.

Pro = domestic wheat production in 1,000 tons.

GDP = per capita GDP in nominal US $.

y82 =Year > 1981 dummy (yes = 1).

The estimated error correction equation (ECT) for India is:


$$\begin{array}{c} {ECT}_{t-1}=lnPC_{t-1}-0.03{△lnIMP}_{t-1}-0.160.3△{ln\%UR}_{t-1}\\ -7.44△lnDPR_{t-1}+56.6△lnGDP_{t-1}\\ -1.82\;\left(Year>1981\;dummy\right)\\ -57.4\;\left(Year>1981\;dummy\;\times △lnGDP_{t-1}\right)\\ +164.2\;\left(Year>1981\;dummy\;\times △{ln\%UR}_{t-1}\right) \end{array}$$

(5) $$\begin{array}{c} +\;0.02 \end{array}$$

And, setting the yearly per capita wheat consumption (PC) as the target variable, the estimated equation for India, is as follows:


$$\begin{array}{c} {△lnPC}_{t}=0.005-0.59{△lnPC}_{t-1}+0.002\;{△lnIMP}_{t-1}\\ +\;0.58{△ln\%UR}_{t-1}-\;0.14{△lnDPR}_{t-1}-0.69{△lnGDP}_{t-1}\\ -0.86\;\left(year>1981\;dummy\right)\\ +0.06\;\left({year>1981\;dummy\times △lnGDP}_{t-1}\right)\\ -0.11\left({year>1981\;dummy\times △ln\%UR}_{t-1}\right) \end{array}$$

(6) $$\begin{array}{c} -0.001 \end{array}$$

From Equation (5), in the long run, with other factors remaining the same, the percentage share of urban population (*p* < 0.00); domestic production (*p* < 0.00), year>1981 dummy (p < 0.00) and the GDP per capita after year 1981 will have a positive impact on yearly per capita wheat consumption in the long run, and conversely, the share of urban population after 1981 will have a negative and significant (*p* < 0.00) impact on India's per capita wheat consumption. It is found that import of wheat has no significant impact on wheat consumption in India.

**Figure 3:**
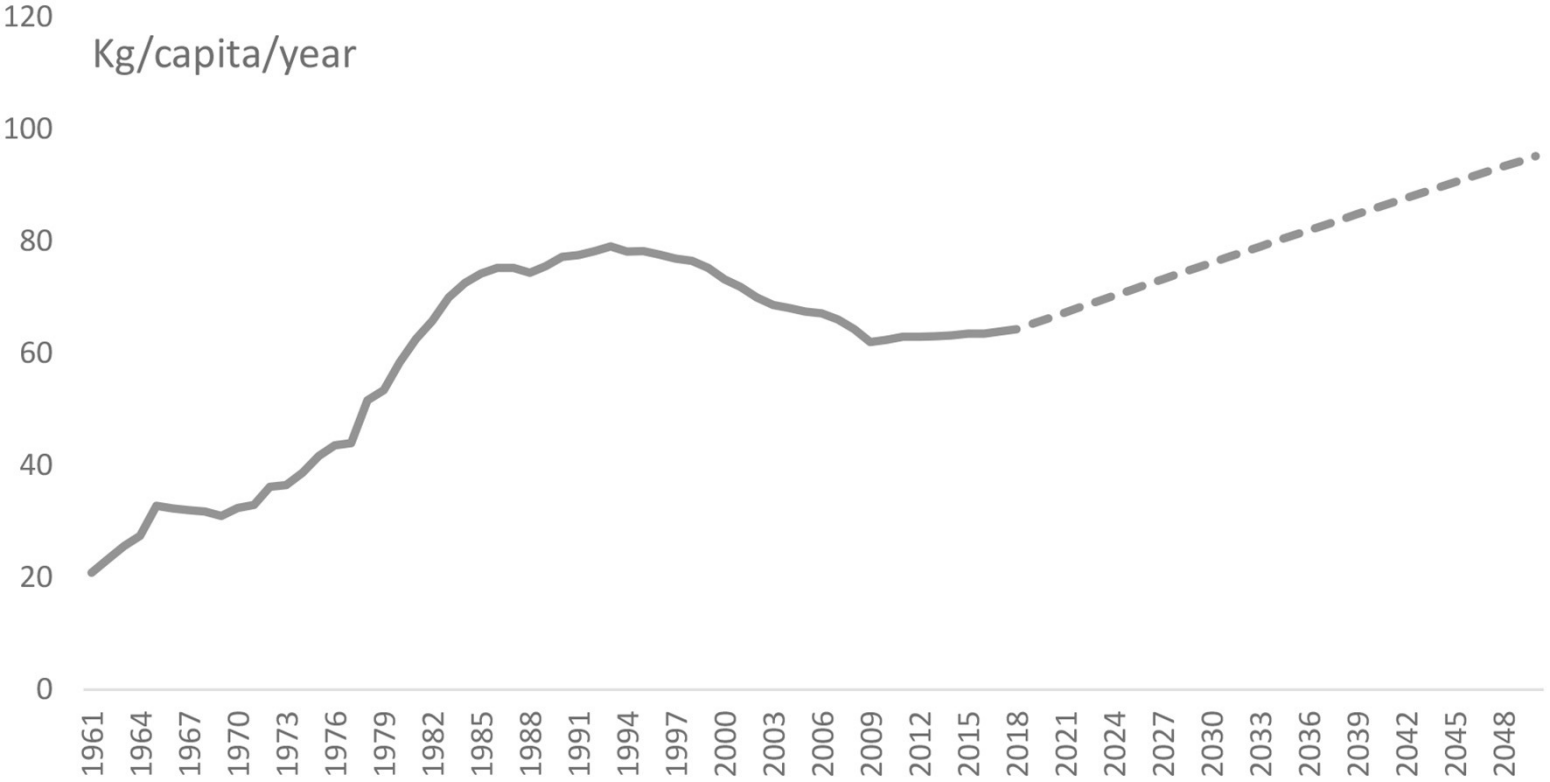
Actual (1961–2018) and predicted (2019–2050) yearly per capita wheat consumption in kg in China, [ln (kg/ per capita /year)], based on the Vector Error Correction (VEC) model estimation procedure. Broken line reflects projected consumption. Source: Authors' estimation.

**Figure 4:**
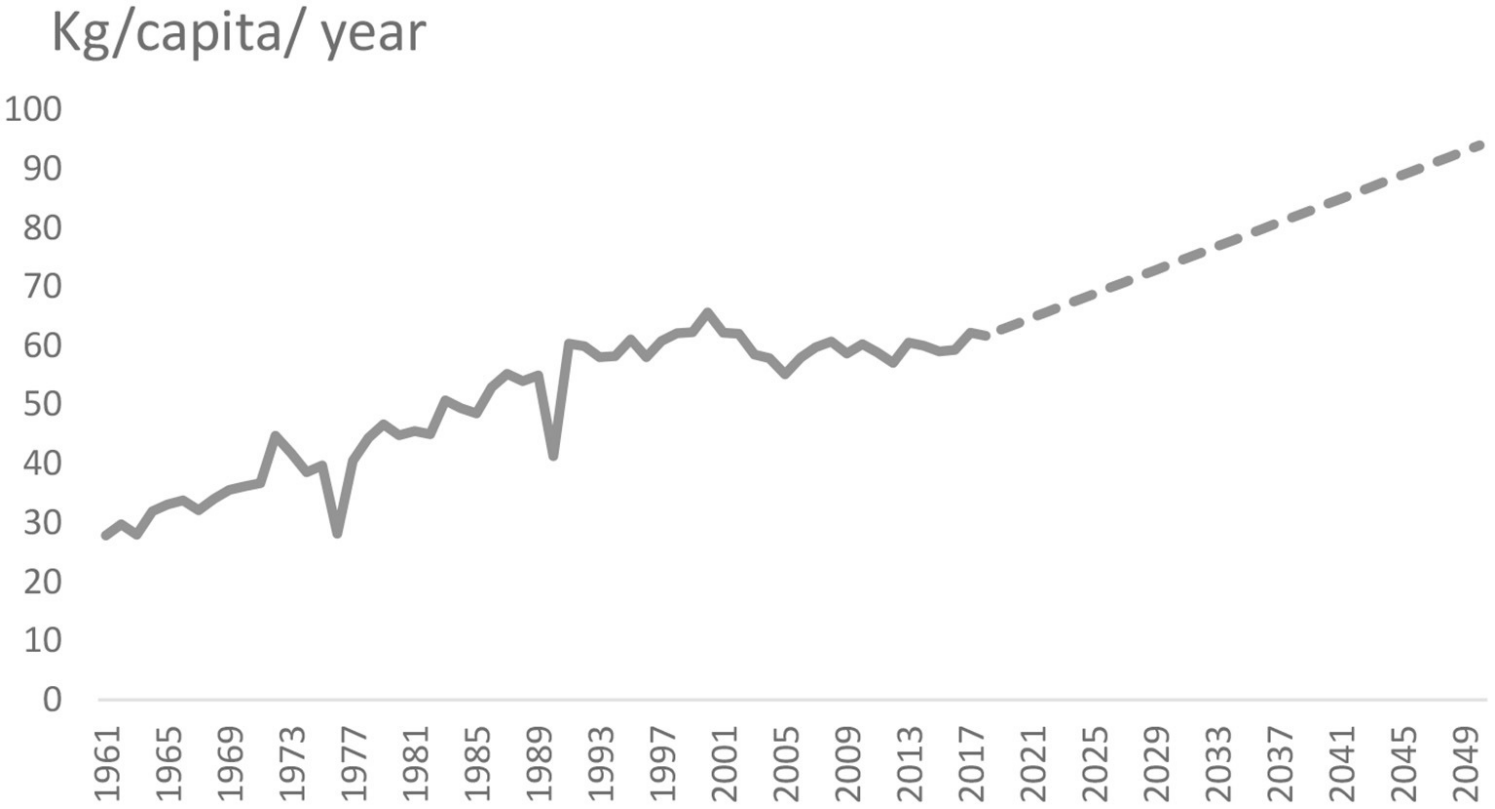
Actual and predicted yearly per capita wheat consumption in KG in India, during 1961–2050 [ln(yearly/capita/KG)], based on Vector Error Correction (VEC) model estimation procedure. Broken line reflects projected consumption. Source: Authors' estimation.

The yearly per capita wheat consumption values in China and India for 2030 and 2050 were forecast using the dynamic forecasting method. Figures 3, 4 present the actual yearly per capita wheat consumption for the period 1961–2018 and the projected yearly per capita wheat consumption of China and India for the period 2019–2050.

The econometric forecasting shows that, by 2030, the yearly per capita wheat consumption in China will increase to 76 kg and by 2050 it will increase to 95 kg, up from the per capita consumption level of 65.8 kg in 2019 (Table 6). Our findings contradict the findings of OECD/FAO (52), which states that by 2028 China's yearly per capita wheat consumption would amount to 62.6 kg, lower than the country's actual yearly per capita wheat consumption of 65.8 kg.

**Table 6:** Wheat consumption projection by considering population dynamics and based on predicted consumption in the sampled countries.

**Country**	**China**	**India**
Kg/capita/year in 2019	65.8	60.4
Total wheat consumed in 2019 (million tons)	96.4	82.5
**Predicted consumption (per capita/kg/yearly)**		
2030	76 (+15.5)	74.0 (+22.5)
2050	95 (+44.3)	94 (+55.6)
**Projected aggregate wheat demand in 2030**		
Low fertility rate assumption	109.2 (+13.2)	108.6 (+32.4)
High fertility rate assumption	113.4 (+17.6)	114.0 (+37.4)
**Projected aggregate wheat demand in 2050**		
Low fertility rate assumption	123.0 (+27.6)	140.0 (+69.7)
High fertility rate assumption	144.0 (+49.4)	168.5 (+104.2)

Source: Authors' estimation.

Values in parenthesis and percentage change compared to the base level of per capita and total wheat consumption in 2019.

Considering the United Nations (20) projected population growth rates, by 2030 China will need to produce between 109 and 113 million t, representing 13–18% more wheat than the current total consumption amount of 96.4 million t in 2019 (Table 6). By 2050, depending on the assumption of the fertility rates, the country will need to supply between 123 and 144 million t of grain, which represents +28% to +49% more wheat than the total consumption of 96.4 million t in 2019 (Table 6). China's current average wheat yield is 5.74 t/ha, with 23.3 million ha of land currently allocated for wheat production (Table 2). To meet the aggregate wheat demand by 2050, considering the high fertility-rate assumption, China will either need to bring 1.78 million ha of new land under wheat production or increase wheat yields to 6.18 t/ha.

For India, the estimation shows that, by 2030 and 2050, yearly per capita wheat consumption in India will increase to 74 kg and 94 kg respectively, compared to per capita annual consumption of 60.4 kg in 2019 (Table 6). In contrast to our projection, OECD/FAO (52) projected that by 2028, the yearly per capita wheat consumption of India would amount to 60.3 kg.

Our projection shows that by 2030 and based on United Nations population projections, India will need to produce 109–114 million t of wheat, 32–37% more than the 82.5 million t currently consumed, and 140–168 million t by 2050, which is 70–104% more than current consumption (Table 6). These findings support the findings of Gandhi et al. (53) and Nagarajan (47), which predicted an increase in wheat demand in India due to increases in income and urbanization. India's current average wheat yield is 3.43 t/ha, with 31.4 million ha of land currently allocated for wheat production (Table 2). India will either need to bring extra 9–18 million ha of new land under wheat production or increase wheat yields to 4.46–5.37 t/ha, to meet the projected domestic for 2050.

It is necessary to mention here is that this study relied on a simple prediction process, using only a few years of observations (1961–2018). Future studies should employ more sophisticated and rigorous estimation and prediction process, such as machine learning approach in big datasets in predicting country specific wheat consumption with more model accuracy and prediction power.

### 4.3. Conclusion and policy implications

The estimation in this study shows that per capita GDP, imports, and domestic production significantly influence yearly per capita wheat consumption in China. Similar to China, domestic production has significant and positive impacts on yearly per capita wheat consumption in India but, in contrast to China, wheat imports have a negative and significant impact on the yearly per capita wheat consumption. Furthermore, in China the percentage share of the urban population (p < 0.00) will have a negative impact on the yearly per capita wheat consumption, whereas in India the share of the urban population has no impact.

Currently, 821 million (10.9%) population of the world face hunger (54). By 2050, the world population is expected to increase to between 8.9 billion and 10.6 billion (20) and with it, the number of hungry people is projected to reach 2 billion, most of whom will hail from the global South. As wheat demand continues to increase in the coming decades, it is imperative to ensure the steady domestic production of wheat in China and India, to help minimize imports by those countries and thereby foster the stability of international wheat markets. This in turn will contribute to stable and affordable wheat grain prices, which will surely benefit the consumers of the wheat importing countries. It will ultimately contribute to ensuring food security and thereby in eliminating global hunger, and in attaining the zero-hunger goal of the United Nations by 2030.

Despite tremendous economic progress, during 2019–21, <2.5% of the total population of China and 16.3% of the total population of India were undernourished (55). Given the reality of limited available agricultural land in China and India and based on our findings, investments in research and development relating to cropping systems of wheat and other major cereals are strongly urged. Harnessing genetic gains and enhancing crop yields can significantly contribute to ensuring food security by feeding burgeoning populations in China and India.

## Data availability statement

The datasets presented in this study can be found in online repositories. The names of the repository/repositories and accession number(s) can be found below: https://data.cimmyt.org/dataset.xhtml?persistentId=hdl:11529/10548840.

## Author contributions

The idea gap, conceptual framework, data cleaning and analysis, literature review, and first draft were prepared by KM. GK, AF, KS, and SL-R contributed to data acquisition, revising and editing the draft, and supervising the data analysis process. All authors contributed to the article and approved the submitted version.
