# Supplementary material for: Projecting wheat demand in China and India for 2030 and 2050: Implications for food security
Source: Front Nutr. 2023 Jan 26;9:1077443. doi: 10.3389/fnut.2022.1077443 (PMC9909467; doi:10.3389/fnut.2022.1077443)
Supplement: Supplementary file 1 [file Data_Sheet_1.docx]

**Supplementary material**

Tests for unit root and structural break

1. China

Variable: ln(consumption)

1. Augmented Dickey-Fuller unit-root test (*dfuller)*

dfuller d.lncon,

Dickey–Fuller test for unit root Number of obs = 56

Variable: D.lncon Number of lags = 0

H0: Random walk without drift, d = 0

Dickey–Fuller

Test -------- critical value ---------

statistic 1% 5% 10%

--------------------------------------------------------------

Z(t) -4.890 -3.572 -2.925 -2.598

--------------------------------------------------------------

MacKinnon approximate p-value for Z(t) = 0.0000.

Explanation: Null hypothesis can be rejected at the 1% level

Variable : ln(import)

dfuller d.lnimp

Dickey–Fuller test for unit root Number of obs = 56

Variable: D.lnimp Number of lags = 0

H0: Random walk without drift, d = 0

Dickey–Fuller

Test -------- critical value ---------

statistic 1% 5% 10%

Z(t) -7.505 -3.572 -2.925 -2.598

MacKinnon approximate p-value for Z(t) = 0.0000.

Explanation: Null hypothesis can be rejected at the 1% level

Variable: ln(share of urban population)

dfuller d.lnur

Dickey–Fuller test for unit root Number of obs = 56

Variable: D.lnur Number of lags = 0

H0: Random walk without drift, d = 0

Dickey–Fuller

Test -------- critical value ---------

statistic 1% 5% 10%

Z(t) -6.336 -3.572 -2.925 -2.598

MacKinnon approximate p-value for Z(t) = 0.0000.

Explanation: Null hypothesis can be rejected at the 1% level

Variable: ln(production)

dfuller d.lnpro

Dickey–Fuller test for unit root Number of obs = 56

Variable: D.lnpro Number of lags = 0

H0: Random walk without drift, d = 0

Dickey–Fuller

Test -------- critical value ---------

statistic 1% 5% 10%

Z(t) -7.569 -3.572 -2.925 -2.598

MacKinnon approximate p-value for Z(t) = 0.0000.

Explanation: Null hypothesis can be rejected at the 1% level

Variable: ln(per capita GDP)

dfuller d.lngdp

Dickey–Fuller test for unit root Number of obs = 56

Variable: D.lngdp Number of lags = 0

H0: Random walk without drift, d = 0

Dickey–Fuller

Test -------- critical value ---------

statistic 1% 5% 10%

Z(t) -5.707 -3.572 -2.925 -2.598

MacKinnon approximate p-value for Z(t) = 0.0000.

Explanation: Null hypothesis can be rejected at the 1% level

Variable : ln(per capita GDP) X Year dummy (>1981=1)

.dfuller d.y82gdp, lags (1)

Augmented Dickey–Fuller test for unit root

Variable: D.y82gdp Number of obs = 55

Number of lags = 1

H0: Random walk without drift, d = 0

Dickey–Fuller

Test -------- critical value ---------

statistic 1% 5% 10%

Z(t) -5.221 -3.573 -2.926 -2.598

MacKinnon approximate p-value for Z(t) = 0.0000.

Explanation: Null hypothesis can be rejected at the 1% level

Variable: ln(share of urban population) X Year dummy (>1981=1)

dfuller d.y82ur

Dickey–Fuller test for unit root Number of obs = 56

Variable: D.y82ur Number of lags = 0

H0: Random walk without drift, d = 0

Dickey–Fuller

Test -------- critical value ---------

statistic 1% 5% 10%

Z(t) -7.454 -3.572 -2.925 -2.598

MacKinnon approximate p-value for Z(t) = 0.0000.

1. Phillip-Perron test for unit root

Variable : ln(per capita consumption)

pperron d.lncon,

Phillips–Perron test for unit root Number of obs = 56

Variable: D.lncon Newey–West lags = 3

H0: Random walk without drift, d = 0

Dickey–Fuller

Test -------- critical value ---------

statistic 1% 5% 10%

--------------------------------------------------------------

Z(rho) -32.476 -19.008 -13.348 -10.736

Z(t) -4.898 -3.572 -2.925 -2.598

--------------------------------------------------------------

MacKinnon approximate p-value for Z(t) = 0.0000.

Variable: ln(import)

pperron d.lnimp,

Phillips–Perron test for unit root Number of obs = 56

Variable: D.lnimp Newey–West lags = 3

H0: Random walk without drift, d = 0

Dickey–Fuller

Test -------- critical value ---------

statistic 1% 5% 10%

Z(rho) -49.848 -19.008 -13.348 -10.736

Z(t) -7.596 -3.572 -2.925 -2.598

MacKinnon approximate p-value for Z(t) = 0.0000.

Variable: ln(share of urban population)

pperron d.lnur,

Phillips–Perron test for unit root Number of obs = 56

Variable: D.lnur Newey–West lags = 3

H0: Random walk without drift, d = 0

Dickey–Fuller

Test -------- critical value ---------

statistic 1% 5% 10%

Z(rho) -57.926 -19.008 -13.348 -10.736

Z(t) -6.591 -3.572 -2.925 -2.598

MacKinnon approximate p-value for Z(t) = 0.0000.

Variable: ln(production)

pperron d.lnpro,

Phillips–Perron test for unit root Number of obs = 56

Variable: D.lnpro Newey–West lags = 3

H0: Random walk without drift, d = 0

Dickey–Fuller

Test -------- critical value ---------

statistic 1% 5% 10%

Z(rho) -55.845 -19.008 -13.348 -10.736

Z(t) -7.574 -3.572 -2.925 -2.598

MacKinnon approximate p-value for Z(t) = 0.0000.

Variable: ln(per capita GDP)

pperron d.lngdp,

Phillips–Perron test for unit root Number of obs = 56

Variable: D.lngdp Newey–West lags = 3

H0: Random walk without drift, d = 0

Dickey–Fuller

Test -------- critical value ---------

statistic 1% 5% 10%

Z(rho) -36.025 -19.008 -13.348 -10.736

Z(t) -5.681 -3.572 -2.925 -2.598

MacKinnon approximate p-value for Z(t) = 0.0000.

Varaible: ln(per capita GDP) X year dummy (>1981=1)

pperron d.y82gdp,

Phillips–Perron test for unit root Number of obs = 56

Variable: D.y82gdp Newey–West lags = 3

H0: Random walk without drift, d = 0

Dickey–Fuller

Test -------- critical value ---------

statistic 1% 5% 10%

Z(rho) -56.347 -19.008 -13.348 -10.736

Z(t) -7.489 -3.572 -2.925 -2.598

MacKinnon approximate p-value for Z(t) = 0.0000.

Variable: ln(share of urban population) X year dummy (>year1981=1)

. pperron d.y82ur,

Phillips–Perron test for unit root Number of obs = 56

Variable: D.y82ur Newey–West lags = 3

H0: Random walk without drift, d = 0

Dickey–Fuller

Test -------- critical value ---------

statistic 1% 5% 10%

Z(rho) -55.645 -19.008 -13.348 -10.736

Z(t) -7.455 -3.572 -2.925 -2.598

MacKinnon approximate p-value for Z(t) = 0.0000.

1. Kwiatkowski-Phillips-Schmidt-Shin test for stationarity

Variable : ln(per capita consumption)

KPSS test for D.lncon

Maxlag = 10 chosen by Schwert criterion

Autocovariances weighted by Bartlett kernel

Critical values for H0: D.lncon is trend stationary

10%: 0.119 5% : 0.146 2.5%: 0.176 1% : 0.216

Lag order Test statistic

0 .16

1 .136

2 .111

3 .0955

4 .0896

5 .0871

6 .0857

7 .0852

8 .0856

9 .0872

10 .0893

Variable: ln(import)

kpss d.lnimp

KPSS test for D.lnimp

Maxlag = 10 chosen by Schwert criterion

Autocovariances weighted by Bartlett kernel

Critical values for H0: D.lnimp is trend stationary

10%: 0.119 5% : 0.146 2.5%: 0.176 1% : 0.216

Lag order Test statistic

0 .0404

1 .0415

2 .0483

3 .0577

4 .0645

5 .0694

6 .0805

7 .0947

8 .101

9 .0913

10 .0886

Variable: ln(share of urban population)

kpss d.lnur

KPSS test for D.lnur

Maxlag = 10 chosen by Schwert criterion

Autocovariances weighted by Bartlett kernel

Critical values for H0: D.lnur is trend stationary

10%: 0.119 5% : 0.146 2.5%: 0.176 1% : 0.216

Lag order Test statistic

0 .275

1 .269

2 .228

3 .194

4 .168

5 .152

6 .138

7 .133

8 .129

9 .127

10 .126

Variable: ln(production)

kpss d.lnpro

KPSS test for D.lnpro

Maxlag = 10 chosen by Schwert criterion

Autocovariances weighted by Bartlett kernel

Critical values for H0: D.lnpro is trend stationary

10%: 0.119 5% : 0.146 2.5%: 0.176 1% : 0.216

Lag order Test statistic

0 .0578

1 .0659

2 .0772

3 .081

4 .08

5 .0839

6 .0973

7 .102

8 .104

9 .106

10 .111

Variable: ln(per capita GDP)

kpss d.lngdp

KPSS test for D.lngdp

Maxlag = 10 chosen by Schwert criterion

Autocovariances weighted by Bartlett kernel

Critical values for H0: D.lngdp is trend stationary

10%: 0.119 5% : 0.146 2.5%: 0.176 1% : 0.216

Lag order Test statistic

0 .092

1 .071

2 .0774

3 .0963

4 .116

5 .12

6 .118

7 .121

8 .125

9 .124

10 .126

Varaible: ln(per capita GDP) X year dummy (>1981=1)

kpss d.y82gdp

KPSS test for D.y82gdp

Maxlag = 10 chosen by Schwert criterion

Autocovariances weighted by Bartlett kernel

Critical values for H0: D.y82gdp is trend stationary

10%: 0.119 5% : 0.146 2.5%: 0.176 1% : 0.216

Lag order Test statistic

0 .0765

1 .078

2 .0792

3 .0806

4 .0823

5 .0841

6 .0862

7 .0886

8 .0915

9 .0946

10 .0977

Variable: ln(share of urban population) X year dummy (>year1981=1)

kpss d.y82ur

KPSS test for D.y82ur

Maxlag = 10 chosen by Schwert criterion

Autocovariances weighted by Bartlett kernel

Critical values for H0: D.y82ur is trend stationary

10%: 0.119 5% : 0.146 2.5%: 0.176 1% : 0.216

Lag order Test statistic

0 .0742

1 .0754

2 .0775

3 .0793

4 .0811

5 .0834

6 .0857

7 .088

8 .0908

9 .0937

10 .0968

1. **Zivot-Andrews Unit Root test allowing for a single break in intercept and/or trend**

Variable: ln(per capita consumption)

zandrews d.lncon,

Zivot-Andrews unit root test for D.lncon

Allowing for break in intercept

Lag selection via TTest: lags of D.D.lncon included =1

Minimum t-statistic -4.212 at 1985 (obs 25)

Critical values: 1%: -5.34 5%: -4.80 10%: -4.58

Variable: ln(import)

zandrews d.lnimp,

Zivot-Andrews unit root test for D.lnimp

Allowing for break in intercept

Lag selection via TTest: lags of D.D.lnimp included =0

Minimum t-statistic -7.784 at 2009 (obs 49)

Critical values: 1%: -5.34 5%: -4.80 10%: -4.58

Variable: ln(share of urban population)

zandrews d.lnur,

Zivot-Andrews unit root test for D.lnur

Allowing for break in intercept

Lag selection via TTest: lags of D.D.lnur included = 2

Minimum t-statistic -6.225 at 1977 (obs 17)

Critical values: 1%: -5.34 5%: -4.80 10%: -4.58

Varaible: ln(production)

zandrews d.lnpro,

Zivot-Andrews unit root test for D.lnpro

Allowing for break in intercept

Lag selection via TTest: lags of D.D.lnpro included = 0

Minimum t-statistic -8.986 at 2004 (obs 44)

Critical values: 1%: -5.34 5%: -4.80 10%: -4.58

Variable: ln(per capita GDP)

zandrews d.lngdp,

Zivot-Andrews unit root test for D.lngdp

Allowing for break in intercept

Lag selection via TTest: lags of D.D.lngdp included = 1

Minimum t-statistic -7.760 at 1982 (obs 22)

Critical values: 1%: -5.34 5%: -4.80 10%: -4.58

Variable: ln(per capita GDP) X year dummy (>year 1981=1)

zandrews d.y82gdp,

Zivot-Andrews unit root test for D.y82gdp

Allowing for break in intercept

Lag selection via TTest: lags of D.D.y82gdp included = 0

Minimum t-statistic -8.138 at 1982 (obs 22)

Critical values: 1%: -5.34 5%: -4.80 10%: -4.58

Variable: ln(share of urban population) X year dummy (>year 1981=1)

zandrews d.y82ur,

Zivot-Andrews unit root test for D.y82ur

Allowing for break in intercept

Lag selection via TTest: lags of D.D.y82ur included = 0

Minimum t-statistic -8.053 at 1982 (obs 22)

Critical values: 1%: -5.34 5%: -4.80 10%: -4.58

1. India

Variable: ln(consumption)

1. Augmented Dickey-Fuller unit-root test (*dfuller)*

dfuller d.lncon,

Dickey–Fuller test for unit root Number of obs = 56

Variable: D.lncon Number of lags = 0

H0: Random walk without drift, d = 0

Dickey–Fuller

Test ------- critical value ---------

statistic 1% 5% 10%

Z(t) -11.309 -3.572 -2.925 -2.598

MacKinnon approximate p-valuefor Z(t) = 0.0000.

Explanation: Null hypothesis can be rejected at the 1% level

Variable : ln(import)

dfuller d.lnimp,

Dickey–Fuller test for unit root Number of obs = 56

Variable: D.lnimp Number of lags = 0

H0: Random walk without drift, d = 0

Dickey–Fuller

Test -------- critical value ---------

statistic 1% 5% 10%

--------------------------------------------------------------

Z(t) -9.967 -3.572 -2.925 -2.598

--------------------------------------------------------------

MacKinnon approximate p-value for Z(t) = 0.0000.

Explanation: Null hypothesis can be rejected at the 1% level

Variable: ln(share of urban population)

dfuller d.lnur,

Dickey–Fuller test for unit root Number of obs = 56

Variable: D.lnur Number of lags = 0

H0: Random walk without drift, d = 0

Dickey–Fuller

Test -------- critical value ---------

statistic 1% 5% 10%

Z(t) -10.987 -3.572 -2.925 -2.598

MacKinnon approximate p-value for Z(t) = 0.0000.

Explanation: Null hypothesis can be rejected at the 1% level

Variable: ln(production)

dfuller d.lnpro,

Dickey–Fuller test for unit root Number of obs = 56

Variable: D.lnpro Number of lags = 0

H0: Random walk without drift, d = 0

Dickey–Fuller

Test -------- critical value ---------

statistic 1% 5% 10%

Z(t) -8.650 -3.572 -2.925 -2.598

MacKinnon approximate p-value for Z(t) = 0.0000.

Explanation: Null hypothesis can be rejected at the 1% level

Variable: ln(per capita GDP)

dfuller d.lngdp,

Dickey–Fuller test for unit root Number of obs = 56

Variable: D.lngdp Number of lags = 0

H0: Random walk without drift, d = 0

Dickey–Fuller

Test -------- critical value ---------

statistic 1% 5% 10%

Z(t) -6.308 -3.572 -2.925 -2.598

MacKinnon approximate p-value for Z(t) = 0.0000.Explanation: Null hypothesis can be rejected at the 1% level

Variable : ln(per capita GDP) X Year dummy (>1981=1)

dfuller d.y82gdp,

Dickey–Fuller test for unit root Number of obs = 56

Variable: D.y82gdp Number of lags = 0

H0: Random walk without drift, d = 0

Dickey–Fuller

Test -------- critical value ---------

statistic 1% 5% 10%

Z(t) -7.490 -3.572 -2.925 -2.598

MacKinnon approximate p-value for Z(t) = 0.0000.

Explanation: Null hypothesis can be rejected at the 1% level

Variable: ln(share of urban population) X Year dummy (>1981=1)

dfuller d.y82ur,

Dickey–Fuller test for unit root Number of obs = 56

Variable: D.y82ur Number of lags = 0

H0: Random walk without drift, d = 0

Dickey–Fuller

Test -------- critical value ---------

statistic 1% 5% 10%

Z(t) -7.515 -3.572 -2.925 -2.598

MacKinnon approximate p-value for Z(t) = 0.0000.

1. Phillip-Perron test for unit root

Variable : ln(per capita consumption)

pperron d.lncon,

Phillips–Perron test for unit root Number of obs = 56

Variable: D.lncon Newey–West lags = 3

H0: Random walk without drift, d = 0

Dickey–Fuller

Test -------- critical value ---------

statistic 1% 5% 10%

Z(rho) -69.287 -19.008 -13.348 -10.736

Z(t) -12.845 -3.572 -2.925 -2.598

MacKinnon approximate p-value for Z(t) = 0.0000.

Variable: ln(import)

pperron d.lnimp,

Phillips–Perron test for unit root Number of obs = 56

Variable: D.lnimp Newey–West lags = 3

H0: Random walk without drift, d = 0

Dickey–Fuller

Test -------- critical value ---------

statistic 1% 5% 10%

Z(rho) -59.510 -19.008 -13.348 -10.736

Z(t) -11.742 -3.572 -2.925 -2.598

MacKinnon approximate p-value for Z(t) = 0.0000.

Variable: ln(share of urban population)

pperron d.lnur,

Phillips–Perron test for unit root Number of obs = 56

Variable: D.lnur Newey–West lags = 3

H0: Random walk without drift, d = 0

Dickey–Fuller

Test -------- critical value ---------

statistic 1% 5% 10%

Z(rho) -61.529 -19.008 -13.348 -10.736

Z(t) -14.973 -3.572 -2.925 -2.598

MacKinnon approximate p-value for Z(t) = 0.0000.

Variable: ln(production)

pperron d.lnpro,

Phillips–Perron test for unit root Number of obs = 56

Variable: D.lnpro Newey–West lags = 3

H0: Random walk without drift, d = 0

Dickey–Fuller

Test -------- critical value ---------

statistic 1% 5% 10%

Z(rho) -65.024 -19.008 -13.348 -10.736

Z(t) -8.648 -3.572 -2.925 -2.598

MacKinnon approximate p-value for Z(t) = 0.0000.

Variable: ln(per capita GDP)

pperron d.lngdp,

Phillips–Perron test for unit root Number of obs = 56

Variable: D.lngdp Newey–West lags = 3

H0: Random walk without drift, d = 0

Dickey–Fuller

Test -------- critical value ---------

statistic 1% 5% 10%

Z(rho) -51.595 -19.008 -13.348 -10.736

Z(t) -6.401 -3.572 -2.925 -2.598

MacKinnon approximate p-value for Z(t) = 0.0000.

Variable: ln(per capita GDP) X year dummy (>1981=1)

pperron d.y82gdp,

Phillips–Perron test for unit root Number of obs = 56

Variable: D.y82gdp Newey–West lags = 3

H0: Random walk without drift, d = 0

Dickey–Fuller

Test -------- critical value ---------

statistic 1% 5% 10%

Z(rho) -55.957 -19.008 -13.348 -10.736

Z(t) -7.492 -3.572 -2.925 -2.598

MacKinnon approximate p-value for Z(t) = 0.0000.

Variable: ln(share of urban population) X year dummy (>year1981=1)

pperron d.y82ur,

Phillips–Perron test for unit root Number of obs = 56

Variable: D.y82ur Newey–West lags = 3

H0: Random walk without drift, d = 0

Dickey–Fuller

Test -------- critical value ---------

statistic 1% 5% 10%

Z(rho) -56.215 -19.008 -13.348 -10.736

Z(t) -7.517 -3.572 -2.925 -2.598

MacKinnon approximate p-value for Z(t) = 0.0000.

1. Kwiatkowski-Phillips-Schmidt-Shin test for stationarity

Variable : ln(per capita consumption)

kpss d.lncon

KPSS test for D.lncon

Maxlag = 10 chosen by Schwert criterion

Autocovariances weighted by Bartlett kernel

Critical values for H0: D.lncon is trend stationary

10%: 0.119 5% : 0.146 2.5%: 0.176 1% : 0.216

Lag order Test statistic

0 .0109

1 .0186

2 .0245

3 .0323

4 .0462

5 .0603

6 .0605

7 .0773

8 .0817

9 .0752

10 .0817

Variable: ln(import)

kpss d.lnimp

KPSS test for D.lnimp

Maxlag = 10 chosen by Schwert criterion

Autocovariances weighted by Bartlett kernel

Critical values for H0: D.lnimp is trend stationary

10%: 0.119 5% : 0.146 2.5%: 0.176 1% : 0.216

Lag order Test statistic

0 .0113

1 .0162

2 .0296

3 .0326

4 .0366

5 .0491

6 .0679

7 .0765

8 .0824

9 .111

10 .107

Variable: ln(share of urban population)

kpss d.lnur

KPSS test for D.lnur

Maxlag = 10 chosen by Schwert criterion

Autocovariances weighted by Bartlett kernel

Critical values for H0: D.lnur is trend stationary

10%: 0.119 5% : 0.146 2.5%: 0.176 1% : 0.216

Lag order Test statistic

0 .0161

1 .026

2 .058

3 .0656

4 .0745

5 .0813

6 .0746

7 .0804

8 .0972

9 .0917

10 .0886

Variable: ln(production)

kpss d.lnpro

KPSS test for D.lnpro

Maxlag = 10 chosen by Schwert criterion

Autocovariances weighted by Bartlett kernel

Critical values for H0: D.lnpro is trend stationary

10%: 0.119 5% : 0.146 2.5%: 0.176 1% : 0.216

Lag order Test statistic

0 .0263

1 .0323

2 .0398

3 .0388

4 .0431

5 .0508

6 .0635

7 .0725

8 .0772

9 .0932

10 .103

Variable: ln(per capita GDP)

kpss d.lngdp

KPSS test for D.lngdp

Maxlag = 10 chosen by Schwert criterion

Autocovariances weighted by Bartlett kernel

Critical values for H0: D.lngdp is trend stationary

10%: 0.119 5% : 0.146 2.5%: 0.176 1% : 0.216

Lag order Test statistic

0 .0244

1 .0288

2 .0375

3 .0445

4 .0651

5 .0876

6 .098

7 .0948

8 .0839

9 .0921

10 .106

Variable: ln(per capita GDP) X year dummy (>1981=1)

kpss d.y82gdp

KPSS test for D.y82gdp

Maxlag = 10 chosen by Schwert criterion

Autocovariances weighted by Bartlett kernel

Critical values for H0: D.y82gdp is trend stationary

10%: 0.119 5% : 0.146 2.5%: 0.176 1% : 0.216

Lag order Test statistic

0 .0703

1 .0718

2 .0736

3 .0756

4 .0777

5 .08

6 .0822

7 .0846

8 .0872

9 .0902

10 .0935

Variable: ln(share of urban population) X year dummy (>year1981=1)

kpss d.y82ur

KPSS test for D.y82ur

Maxlag = 10 chosen by Schwert criterion

Autocovariances weighted by Bartlett kernel

Critical values for H0: D.y82ur is trend stationary

10%: 0.119 5% : 0.146 2.5%: 0.176 1% : 0.216

Lag order Test statistic

0 .0706

1 .0724

2 .0744

3 .0765

4 .0783

5 .0803

6 .0826

7 .0852

8 .0876

9 .0904

1. .0935

d. Zivot-Andrews Unit Root test allowing for a single break in intercept and/or trend

Variable: ln(per capita consumption)

zandrews d.lncon,

Zivot-Andrews unit root test for D.lncon

Allowing for break in intercept

Lag selection via TTest: lags of D.D.lncon included = 0

Minimum t-statistic -11.663 at 1977 (obs 17)

Critical values: 1%: -5.34 5%: -4.80 10%: -4.58

Variable: ln(import)

zandrews d.lnimp,

Zivot-Andrews unit root test for D.lnimp

Allowing for break in intercept

Lag selection via TTest: lags of D.D.lnimp included = 1

Minimum t-statistic -9.533 at 2000 (obs 40)

Critical values: 1%: -5.34 5%: -4.80 10%: -4.58

Variable: ln(share of urban population)

zandrews d.lnur,

Zivot-Andrews unit root test for D.lnur

Allowing for break in intercept

Lag selection via TTest: lags of D.D.lnur included = 1

Minimum t-statistic -11.928 at 1984 (obs 24)

Critical values: 1%: -5.34 5%: -4.80 10%: -4.58

Variable: ln(production)

zandrews d.lnpro,

Zivot-Andrews unit root test for D.lnpro

Allowing for break in intercept

Lag selection via TTest: lags of D.D.lnpro included = 0

Minimum t-statistic -8.899 at 1973 (obs 13)

Critical values: 1%: -5.34 5%: -4.80 10%: -4.58

Variable: ln(per capita GDP)

zandrews d.lngdp,

Zivot-Andrews unit root test for D.lngdp

Allowing for break in intercept

Lag selection via TTest: lags of D.D.lngdp included = 1

Minimum t-statistic -7.378 at 1972 (obs 12)

Critical values: 1%: -5.34 5%: -4.80 10%: -4.58

Variable: ln(per capita GDP) X year dummy (>year 1981=1)

zandrews d.y82gdp,

Zivot-Andrews unit root test for D.y82gdp

Allowing for break in intercept

Lag selection via TTest: lags of D.D.y82gdp included = 0

Minimum t-statistic -8.015 at 1982 (obs 22)

Critical values: 1%: -5.34 5%: -4.80 10%: -4.58

Variable: ln(share of urban population) X year dummy (>year 1981=1)

zandrews d.y82ur,

Zivot-Andrews unit root test for D.y82ur

Allowing for break in intercept

Lag selection via TTest: lags of D.D.y82ur included = 0

Minimum t-statistic -8.043 at 1982 (obs 22)

Critical values: 1%: -5.34 5%: -4.80 10%: -4.58
